# USP4 function and multifaceted roles in cancer: a possible and potential therapeutic target

**DOI:** 10.1186/s12935-020-01391-9

**Published:** 2020-07-10

**Authors:** Yizhi Wang, Li Zhou, Jun Lu, Bolun Jiang, Chengxi Liu, Junchao Guo

**Affiliations:** Department of General Surgery, Peking Union Medical College Hospital, Chinese Academy of Medical Sciences and Peking Union Medical College, Beijing, 100730 China

**Keywords:** USP4, Post-translational modification, Cancer, Mechanisms, Therapeutic target

## Abstract

Cancer remains one of the major culprits causing disease-related deaths and leads to a high morbidity and similar mortality. Insidious onset, difficult early detection and a lack of broad-spectrum and effective multi-cancer therapeutic targets have limited the prolongation of cancer patients’ survival for decades. Therefore, a versatile therapeutic target which is involved in various cancer-related signaling pathways and different cancers may be more effective for cancer targeted therapy. USP4, one of the DUBs members which participates in deubiquitination, an inverse process of ubiquitination, can regulate various classical cancer-related signaling pathways, and thereby plays a vital role in some pathological and physiological processes including tumor initiation and progression. Recently, USP4 has been found to exert versatile influences on cells proliferation, migration and invasion, also apoptosis of various tumors. Moreover, USP4 can also act as a prognostic biomarker in several cancers. This review will give a comprehensive introduction of USP4 about its regulatory mechanisms, related signaling pathways, pathophysiological functions and the roles in various cancers which may help us better understand its biological functions and improve future studies to construct suitable USP4-targeted cancer therapy system.

## Background

The morbidity of cancer has been increasing recent years with the improvement of life quality and life expectancy. It is estimated that people suffering from cancers will increase to over 29.5 million globally by 2040 [1]. The occurrence of cancers can cause great financial burden and psychologic stress to both individuals and the whole society due to various factors, such as lower income, unemployment and the time-consuming and inefficiency adjuvant and palliative therapy, especially to younger and socioeconomically disadvantaged patients [2, 3]. Insidious onset, early metastasis and scarce effective targeted drugs may cause tumor refractory and recurrence, especially in lung cancer and pancreatic cancer, which can lead to a higher mortality [4, 5]. Radical surgery is an ideal therapy for cancer control, however, limited patients are suitable for it. And existing targeted therapies are too hard to obtain satisfactory results [6, 7]. Therefore, novel therapeutic targets are urgently needed to broaden future methods of cancer treatment.

Post-translational modifications (PTMs) play various roles in proteins expression and function modulation without changing mRNAs expression level or even total proteins expression level which mainly include phosphorylation, SUMOylation, glycosylation, ubiquitination, acetylation and some uncommon PTMs such as lipidation, citrullination [8–14]. PTMs are involved in various physiological and pathological processes including cancers due to countless targeted proteins. Therefore, proteins possessing PTMs capacity have always been deemed as potential and promising therapeutic targets for cancer treatment [15, 16].

Ubiquitination, a member of PTMs, can regulate proteins expression and function through attaching ubiquitin, a 76aa-protein, to the targeted proteins through the sequential activation of the ubiquitin-activating enzyme, ubiquitin-conjugating enzyme and ubiquitin ligase which lead to either their stabilization or inactivation, then degradation through proteasomal pathway [17, 18]. Ubiquitination is a complicated process which requires several components to participate jointly. Therefore, mistakes in any part can result in failure of ubiquitination accomplishment which can be used as therapeutic targets for diseases treatment [19].

Deubiquitination is the inverse process of ubiquitination which is mediated by deubiquitylating enzymes (DUBs). In the process of deubiquitination, ubiquitin can be removed from the substrates and then prevent substrates from stabilization or degradation which depends on the detailed deubiquitination sites [20]. DUBs consist five subclasses, namely, metalloproteinase, ubiquitin C-terminal hydrolases, Machado–Joseph disease proteins, otubain proteases and ubiquitin-specific proteases (USPs) [20]. USPs account for the largest proportion in DUBs which contain nearly 70 members in human species [21]. The main function of USPs is to cleave linear and/or branched ubiquitin precursor from monoubiquitinated or polyubiquitinated substrates and then stabilize or inactivate target proteins [22]. As main members of the deubiquitinase family, many studies have explored the role of USPs in various diseases, especially in cancer. Due to numerous substrates of USPs, USPs can exert versatile functions in tumor progression, including epithelial–mesenchymal transition (EMT) and stemness of cancer, metastasis, tumor-association microenvironment and DNA damage repair activity [23]. Therefore, because most USPs can positively regulate cancer progression, many inhibitors have been developed to target the specific sites in USPs to hamper this process, such as broad-spectrum inhibitors WP1130 and PR619 [24, 25]. However, development of specific and selective USPs inhibitor is difficult due to the conserved domain in USPs.

Ubiquitin-specific protease 4 (USP4), which is located in chromosome 3 (3p21,3), is one of the USPs family which can regulate various signaling pathways by deubiquitinating vital proteins [26]. Like other USPs, previous basic experimental studies have unmasked the versatile roles of USP4 in numerous pathological and physiological processes, especially in cancers. However, to the best of our knowledge, compared with other USPs, USP4 has not been systematically and specifically reviewed so far. And the cancer target potential and clinical application value of USP4 may be underestimated. Therefore, the present review will give a comprehensive overview about its regulatory mechanisms, involved signaling pathways, roles in pathological and physiological process, especially in different cancers to raise further studies of USP4 in extensive cancer therapy and clinical application.

### Regulatory mechanisms of USP4 protein function

Baker et al. confirmed that USP4 was a nucleocytoplasmic shuttling protein which can locate both in the cytoplasm and nucleus. However, the accumulation of USP4 expression in the cytoplasm and nucleus varies among different cell types [27]. USP4 phosphorylation is an indispensable process leading to the localization of USP4 from nucleus to cytoplasm. Phosphorylated USP4 was found in cytoplasm and cell membrane in breast cancer cells. Further study showed that USP4 can be phosphorylated at its Ser 445 by activated protein kinase B (AKT) and bind to 14-3-3 isoforms to be exported from nucleus to cytoplasm and subsequently exert its deubiquitination capacity [28]. Moreover, AKT-mediated USP4 phosphorylation can abolish the ubiquitination of Rheb and then stabilize it, which activate the mechanistic target of rapamycin complex 1-mediated signaling pathways, thus influenced tumor growth [29]. In addition, cyclin-dependent kinases (CDKs) were also involved in USP4 phosphorylation which can be hampered by purvalanol A, a CDKs inhibitor. Dephosphorylation of USP4 can cause its nuclear accumulation which enhances its interaction with squamous cell carcinoma antigen recognized by T cells 3 (SART3) and regulate spliceosome dynamics through deubiquitinating precursor RNA processing 3 [30]. And SART3 can also improve the deubiquitinated activity of USP4 on K63-linked polyubiquitin chains of RNA-binding protein with serine-rich domain 1, and stabilize it to form the spliceosome and activate the pre-mRNA splicing process [31]. Besides deubiquitinating polyubiquitin chains to stabilize targeted proteins, USP4 was reported to inhibit the mono-ubiquitination of phosphor-inositide-dependent kinase 1 which enhanced its activation to promote cell proliferation and metabolism [32]. In addition, USP4 can also interact with the S9/Rpn6 subunit of the proteasome and regulate its function without knowing the detailed mechanisms [33]. These studies imply that the role of USP4 may be context-dependent.

### Regulated signaling pathways

**Fig. 1:**
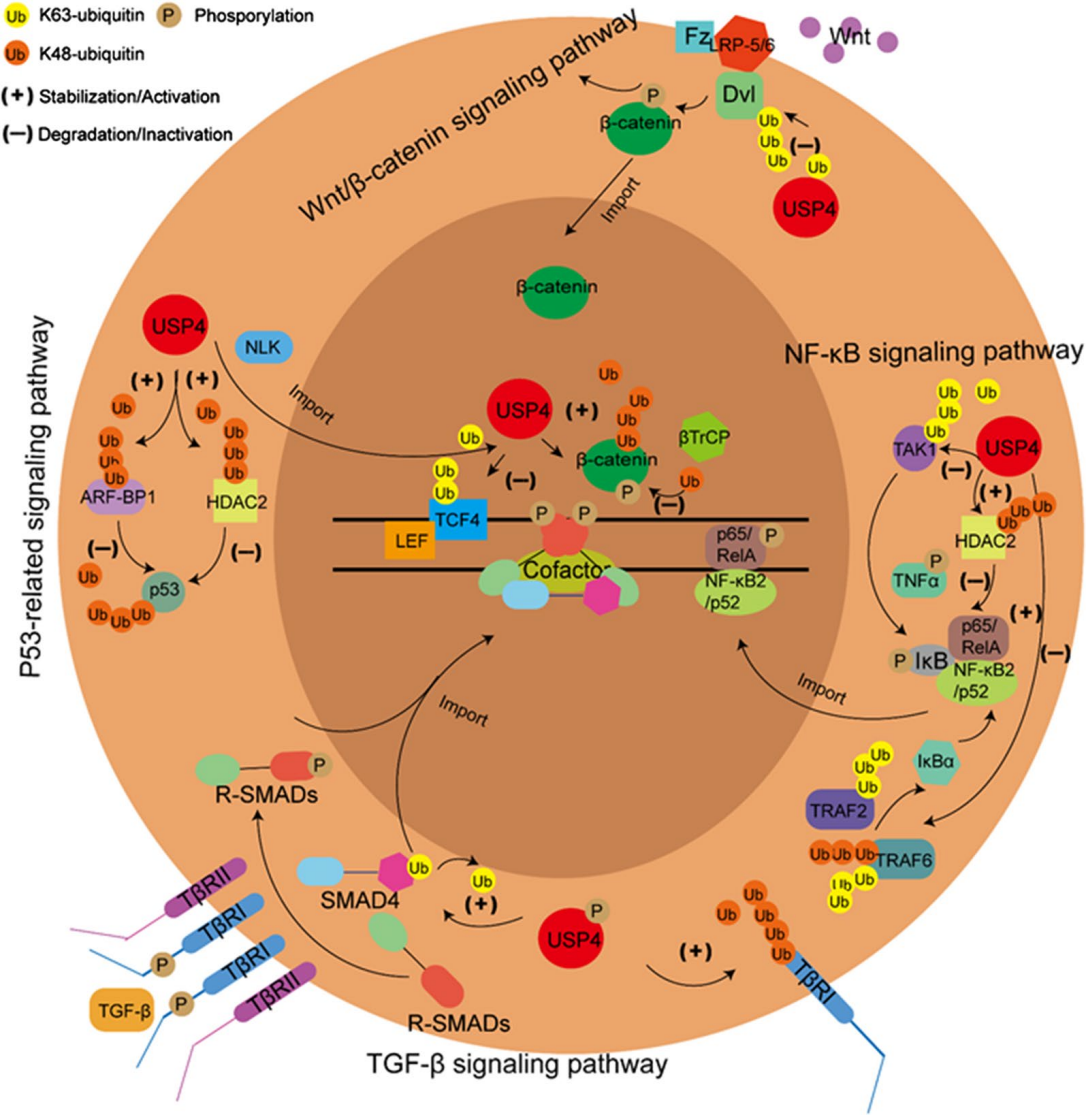
Vital signaling pathways and important substrates for downstream regulation of USP4. In Wnt/β-catenin signaling pathway, USP4 can stabilize β-catenin by deubiquitinating its K48-ubiquitin and inactivate Dvl and TCF4 by deubiquitinating their K63-ubiquitin. In TGF-β signaling pathway, USP4 can stabilize TβRI by deubiquitinating its K48-ubiquitin and deubiquitinate SMAD4 mono-ubiquitination. USP4 can inactivate TAK1, TRAF2 and TRAF6 and stabilize HDAC2 to inhibit NF-κB signaling pathway. Also, USP4 can stabilize TRAF6 to activate NF-κB signaling pathway. In p53-related signaling pathway, USP4 can stabilize ARF-BP1 and HDAC2 by deubiquitinating its K48-ubiquitin to promote the ubiquitination and degradation of p53

USP4 has been reported to be involved in several classical and vital signaling pathways due to various substrates, such as canonical and noncanonical Wnt/β-catenin signaling pathway, canonical and noncanonical tumor growth factor-β (TGF-β) signaling pathway, nuclear factor kappa B (NF-κB) signaling pathway and also p53-related signaling pathway, which modulate numerous pathological and physiological processes [34]. The overview of the roles of USP4 in signaling pathways regulation is shown in Fig. 1.

### Wnt/β-catenin signaling pathway

In canonical Wnt/β-catenin signaling pathway, β-catenin is dephosphorylated by Wnt-induced the Frizzled surface receptor activation to enhance its nuclear translocation and accumulation. Then activated β-catenin in turn activates downstream transcriptional factors, T-cell factor (TCF) and lymphoid enhancer factor (LEF) to further regulate the targeted genes [35, 36]. USP4 was once found to accumulate in nucleus after Nemo-like kinase (NLK) activation and target TCF4 to inhibit its transcriptional activity, whereby inactivate Wnt/β-catenin signaling pathway [37]. Additionally, phosphorylated β-catenin can be recognized by multi-subunit ubiquitin ligase, F-box protein βTrCP, to promote subsequent ubiquitination and proteasomal degradation [38]. USP4 also showed an positive role in Wnt/β-catenin signaling pathway regulation by deubiquitinating and stabilizing β-catenin which also facilitated its nuclear transcriptional regulation capacity [38].

### TGF-β signaling pathway

TGF-β signaling pathway can be divided into canonical TGF-β/Smad signaling pathway and noncanonical non-Smad pathway [39]. As aforementioned, phosphorylated USP4 can relocate from nucleus to cytoplasm or cell membrane where USP4 deubiquitylates TGF-β receptor I (TβRI) and stabilizes its expression to activate TGF-β signaling pathway to induce R-Smads phosphorylation and then R-Smads are imported into the nucleus to regulate targeted genes expression [28]. Moreover, Smad4 can be inhibited through inhibitory mono-ubiquitination linked by the recruitment of E3 ligase, Smurf2 which has a competing relationship with USP4. USP4 can participate in this pathway by targeting mono-ubiquitinated Smad4 for deubiquitination and further activating downstream activin/bone morphogenetic protein signaling pathway [40].

### NF-κB signaling pathway

USP4 has usually been a NF-κB signaling pathway suppressor through exerting deubiquitination on upstream regulatory proteins of this signaling pathway. K63-linked polyubiquitination of transforming growth factor-β-activated kinase 1 (TAK1) has been reported to mediate the activation of tumor necrosis factor α (TNFα) and then NF-κB signaling pathway through activating IκB kinase. However, this process can be reversed by the deubiquitination role of USP4 [41]. Besides, doxorubicin-induced NF-κB activation is one of the reasons causing doxorubicin resistance. Further study indicated that TAK1 polyubiquitination at lysine 158 residue was involved in this process which can be reversed by exogenous USP4 administration [42]. TNFα-induced NF-κB activation can also be impeded by exogenous USP4 expression through targeting TNF receptor associated factor (TRAF) 2 and TRAF6 polyubiquitination and promoting downstream NF-κBα inhibitor, IκBα, expression [43]. Moreover, histone deacetylase 1/2 (HDAC1/2) complex can interact with p65 (RelA) subunit which is involved in the transactivation of NF-κB signaling pathway. Therefore, USP4 can also play as a suppressor of NF-κB signaling pathway through deubiquitylating and stabilizing HDAC2 [44]. However, whether USP4 have NF-κB signaling pathway inactivation role or USP4 can directly target components of NF-κB complex to inhibit its activation like its analogue, USP15, still remains to be explored [45].

### P53-related signaling pathway

P53 is well-known for its tumor-suppressing role which also can be ubiquitinated and degradation by various ubiquitin ligases, such as murine double minute 2, coat protein 1, p53-induced protein with a RING-H2 domain and ARF-binding protein 1 (ARF-BP1) in tumor tissues [46–49]. Meanwhile, these ubiquitin ligases themselves can also undergo ubiquitination. USP4 was reported to target ARF-BP1 for deubiquitination and stabilization then enhance its ubiquitination capacity to induce p53 degradation which indicated its tumor-promoting role [50]. HDAC2 was also indicated to be involved in the modulation of USP4 to p53 inactivation [44]. Generally speaking, USP4 plays as a tumor promoter through p53 regulation.

### USP4 in pathological and physiological modulation

Given numerous substrates and pivotal signaling pathways influenced by USP4, USP4 has been reported to modulate various human pathological and physiological processes which is concluded in Table 1.

**Table 1:** The pathophysiologic processes modulated by USP4

Authors, year	Pathophysiologic processes	Deubiquitinated targets	Effects to the deubiquitinated targets	Detailed mechanisms	Refs.
Zhu et al., 2018	Liver fibrosis	TβRI	Stabilization	HSC and EMT activation; liver fibrosis enhancement	[51]
Zhao et al., 2018	NAFLD pathogenesis	TAK1	Inactivation	Alleviation NAFLD progression through inactivation of NF-κB and JNK signaling pathways	[53]
Zhou et al., 2019	Hepatic I/R injury	TAK1	Inactivation	Alleviation hepatic I/R injury by inhibiting inflammatory caused by NF-κB signaling pathway	[54]
Zhou et al., 2012	Innate immune response and immune homeostasis	TRAF6	Inactivation	Inhibition of the immunoinflammatory response caused by TLR/IL-1R-induced NF-κB activation	[56]
Yang et al., 2015	Th17 cell function	RORγt	Stabilization	IL-17 expression upregulation	[57]
Lin et al., 2017	Treg cell function	IRF8	Stabilization	Enhancement of IL-10, TGF-β expression to promote Treg cell function	[60]
Guo et al., 2017	Th2 cell function	IRF4	Stabilization	Enhancement of IL-4 expression to promote Th2 cell function	[61]
Jiang et al., 2017	Spinal cord injury	TRAF6	Inactivation	Inhibition of secondary spinal cord injury through NF-κB inactivation	[63]
Wang et al., 2013	Antiviral response	RIG-I	Stabilization	Activation of RIG-I-induced IFN-β signaling pathway and VSV replication inhibition	[70]
Xu et al., 2018	Antiviral response	TRAF6	Stabilization	Activation of TRAF6-mediated NF-κB signaling pathway and EV 71 replication inhibition	[71]
Wijinhoven et al., 2015	DNA breaks repairment	USP4	Stabilization	DNA repairment enhancement	[73]
Zhou et al., 2016	Osteoblast differentiation	Dvl	Inactivation	Inhibition of Wnt/β-catenin signaling pathway mediated osteoblast differentiation	[80]
Yun et al., 2017	Myogenesis	HDAC1 and 4	Stabilization	Enhancement of MyoD to inhibit myogenesis	[82]
He et al., 2016	Pathological cardiac hypertrophy	TAK1	Inactivation	Inactivation of TAK1/JNK1/2/p38 to inhibit pathological cardiac hypertrophy	[83]

### Hepatocyte lesions

Liver fibrosis is a pathological process after liver damage characterized by accumulation of extracellular matrix secreted by hepatic stellate cells (HSCs) deposited around liver parenchyma [51]. The activation of HSCs and epithelial–mesenchymal transition (EMT) of hepatocyte is stimulated by TGF-β signaling pathway. In fibrotic liver tissues, USP4 was released through overexpression of lncRNA H19 sponging miR-148a and stabilized TβRI expression through deubiquitination to activate TGF-β signaling pathway which induced liver fibrosis [52]. In addition, liver X receptor-α acts as a protective protein to prevent TGF-β-induced liver fibrosis through enhancing downstream cannabinoid receptor 2 transcription and miR-27b rather than miR-148a expression, thereby hinders USP4 expression and inactivates TGF-β signaling pathway [53]. Therefore, both lncRNA H19 and USP4 can be used as targets to liver fibrosis treatment which may be the precancerous lesions of liver cancer. The study of Zhao et al. indicated that USP4 expression significantly decreased in liver tissues from nonalcoholic fatty liver disease (NAFLD) patients. USP4 deficiency in mice hepatocytes exacerbated hepatic steatosis, insulin resistance and inflammatory response induced by high fat diet. Mechanistically, ectopic administration of USP4 directly targeted to deubiquitinate TAK1 and suppressed the activation of NF-κB signaling pathway which ameliorate the extent of fatty liver [54]. This study implies a potential drug role of USP4 in fatty liver therapy. And USP4 deficiency can also aggravate TAK1 signaling pathway-induced hepatic ischaemia/reperfusion injury [55].

### Immunoregulation

The roles of USP4 in immunoregulation have been explored in previous studies. The activation of Toll-like receptor (TLR)/IL-1 receptor (IL-1R) signaling pathway which is mediated by k63-linked polyubiquitinated TRAF6 is responsible for innate immune response and immune homeostasis by regulating downstream immunoregulatory genes through transcriptional factors, NF-κB and activator protein-1 [56]. However, deficiency of activated TRAF6 can cause insufficiency of immune response which results from deubiquitination capacity of endogenous USP4 which leads to TRAF6 inactivation [57]. USP4 is also vital in promoting Th17 immune cells differentiation through deubiquitinating and stabilizing transcriptional factor retinoic acid-related orphan receptor γt (RORγt). Knockdown of USP4 impaired TGF-β and IL-6-mediated Th17 differentiation [58]. Therefore, USP4 can be used as a target to inhibit Th17-mediated autoimmune disease. Subsequently, the same team also reported that in Foxp3+ CD4+ CD25+ regulatory T cells, interferon regulatory factor (IRF) 8, a transcriptional factor, is pivotal for the immunosuppressive function of Treg cells by enhancing the secretion of anti-proinflammatory cytokines such as IL-10, TGF-β [59, 60]. USP4 can deubiquitinate k48-linked IRF8 polyubiquitination and stabilize its expression. On the contrary, depletion of USP4 abolished the immunosuppressive function of Treg on effector T cells [61]. Therefore, anti-USP4 therapy can play a role in overturning immunosuppressive microenvironment of cancers. In the study of rheumatic heart disease, Guo et al. revealed that USP4 positively modulated IL-4 secretion by T helper type 2 cells through deubiquitinating and stabilizing IRF4 which is usually the reason of allergic immune responses diseases, such as rheumatic heart disease [62].

### Neural injury

Due to the inflammatory modulation capacity of USP4, previous studies have discovered the role of USP4 after neural injury. Early study implied that the expression of USP4 was upregulated in neurons adjacent to hematoma after intracerebral hemorrhage (ICH). Further study showed that USP4 was co-expressed with apoptosis-related proteins such as active caspase-3, γH2AX and Bax which indicated USP4 may join in the neural apoptosis modulation after ICH. However, the detailed deubiquitinated substrates and mechanisms still remain to be elucidated [63]. In a subsequent study, Jiang et al. found that USP4 can inhibit the inflammatory response induced by NF-κB signaling pathway in secondary injury after spinal cord injury through deubiquitinating and stabilizing NF-κB signaling pathway upstream polyubiquitinated TRAF2 and 6. Therefore, ectopic USP4 administration may be seemed as a novel therapy for control secondary spinal cord injury [64].

### Antiviral roles

Like other members of ubiquitin-specific proteases, USP4 has also been reported to exert a vital role in antiviral response due to its role in immune response [65, 66]. Retinoic acid-inducible gene I (RIG-I) is an important pattern recognition receptor which can recognize various viruses [67]. After virus recognition, caspase recruitment domain (CARD) of RIG-I can bind to mitochondrial antiviral signaling protein to mediated its oligomerization which enhance IRF3 transcription and the generation of interferon-γ (IFN-γ) [68]. K63-linked polyubiquitination of CARD can induce RIG-I activation while K48-linked polyubiquitination of it can lead to its degradation. Several USPs can modulate both K63 and K48-linked polyubiquitination while USP4 can interact with RIG-I and target its K48-linked polyubiquitination and stabilize its expression to upregulate IFN-β expression then modify antiviral innate immune responses [69–71]. NF-κB signaling pathway has also been reported in antiviral process through stimulating inflammatory responses. K48-linked polyubiquitinated TRAF6 can mediate its degradation which is opposed to the activation function of K63-linked TRAF6 polyubiquitination. Xu et al. suggested that the expression of USP4 was significantly inhibited when enterovirus 71 (EV71) infected. Overexpression of USP4 can target K48-linked polyubiquitination of TRAF6 for deubiquitinating which mediates the EV71 replication suppression caused by the activation role of another pattern recognition receptor, retinoic acid-inducible gene I-like receptor (RLR) to NF-κB signaling pathway [72]. The aforesaid study implies that USP4 possesses the deubiquitination capacity of both K48- and K63-linked polyubiquitination which generate quite opposite effects. The antiviral effect may make USP4 a potential target for virus-induced cancers, such as cervical cancer. However, the detailed effect of USP4 in cervical cancer should be further explored.

### DNA damage repair capacity

DNA double-strand breaks repairment is crucial for maintaining genome integrity which is partially dependent on PTMs such as ubiquitination. USP4 was found to enhance DNA double-strand breaks repairment, DNA-end resection and homologous recombination via a conversed and specific domain to form a complex with CtIP and the MRE11-RAD50-NBS1 complex which can be abrogated by USP4 auto-deubiquitination on several specific cysteine residues. Since USP4 was once reported to have auto-deubiquitination capacity, the role of USP4 in DNA repairment may rely heavily on this function [73, 74]. The DNA repairment capacity may make USP4 a tumor promoter in cancer progression which may be a potential target in tumor therapy.

### Connective tissue development

The unique part of USP4 in the development of some connective tissues has gradually been explored in several studies. Activation of Wnt/β-catenin signaling pathway has long been recognized to enhance osteoblastogenesis and bone formation through modulating various downstream targets while the inactivation of Wnt/β-catenin signaling pathway may lead to a series of bone metabolic diseases [75–80]. Rather than targeting TCF or β-catenin in Wnt/β-catenin signaling pathway, USP4 exerted a negative effect on this pathway by deubiquitinating K63-linked polyubiquitinated Dishevelled (Dvl) which abrogated the formation of a signaling complex and maintained the cytoplasmic accumulation of β-catenin, then hampered the whole pathway in the nucleus [81]. This procedure leads to the inclination to osteoclastogenesis and cause several bone metabolic diseases which is why USP4 can be the target for bone metabolic diseases treatment.

In myogenesis, myotube formation, proliferation, differentiation of myoblasts is strictly and negatively regulated by numerous myogenic regulatory factors including MyoD [82]. Yun’s team revealed a versatile role of USP4 in myogenesis inhibition through its catalytic activity stabilizing HDAC1 and HDAC4 to form a complex with MyoD and enhancing its inhibitory role. Besides, AKT and p38 signaling pathways were also involved in this process without knowing the detailed mechanisms [83]. Besides, the myogenesis suppressive role of USP4 was also found to ameliorate angiotensin II-mediated pathological cardiac hypertrophy both in vitro and in vivo. USP4 expression can deubiquitinate and inhibit the activation of polyubiquitinated TAK1 which hinder the activation of TAK1/c-Jun N-terminal kinase 1/2 (JNK1/2)/p38 signaling pathway [84]. These results indicated the therapeutic potential of USP4 in cardiac hypertrophy treatment.

### Roles of USP4 in cancer

**Fig. 2:**
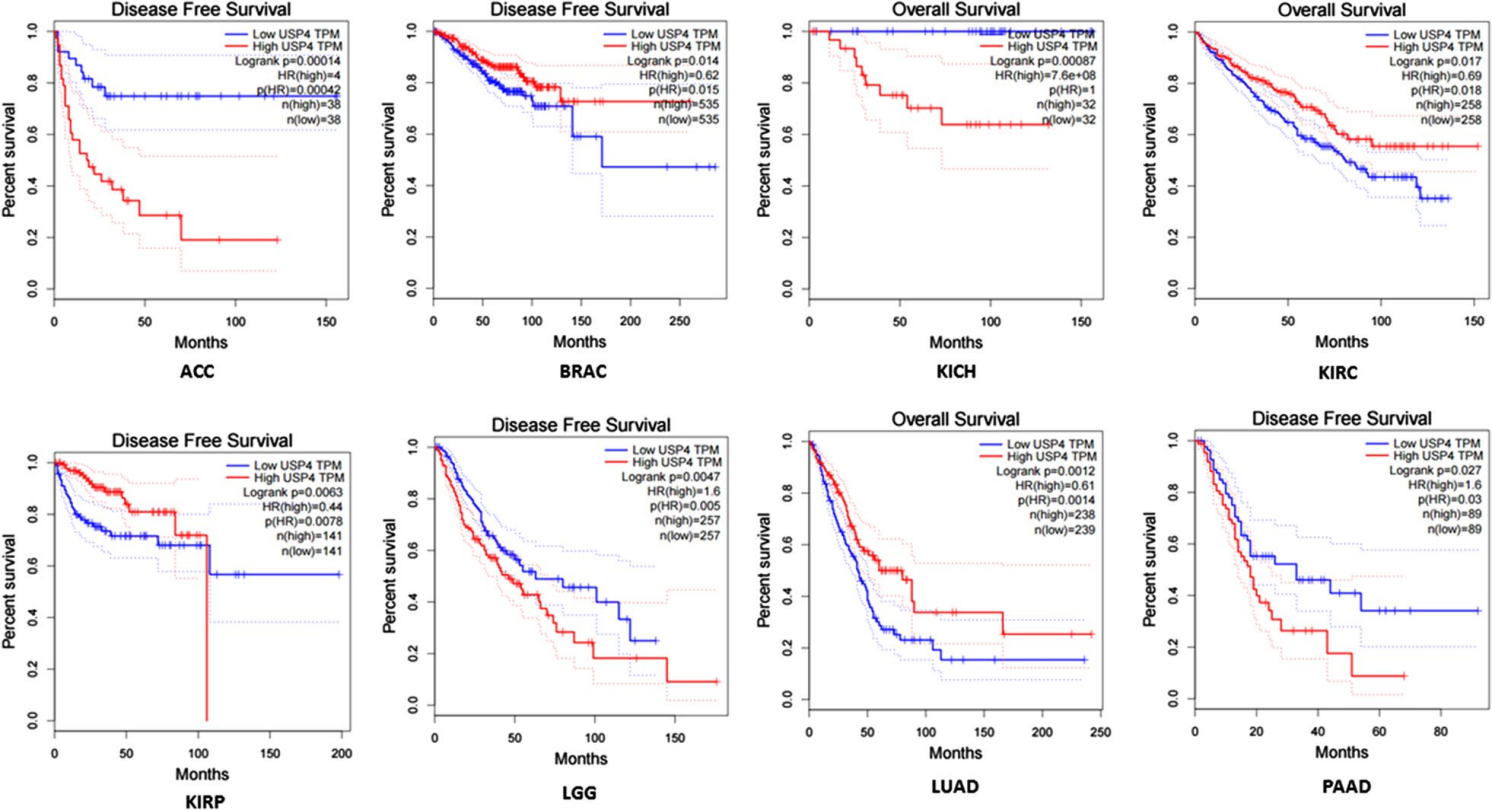
Survival curve of several cancers using Kaplan–Meier method based on TCGA database. High USP4 expression predicts unfavorable prognosis in ACC (unfavorable DFS), KICH (unfavorable OS), LGG (unfavorable DFS) and PAAD (unfavorable DFS) and favorable prognosis in BRAC (favorable DFS), KIRC (favorable OS), KIRP (favorable DFS) and LUAD (favorable OS)

**Fig. 3:**
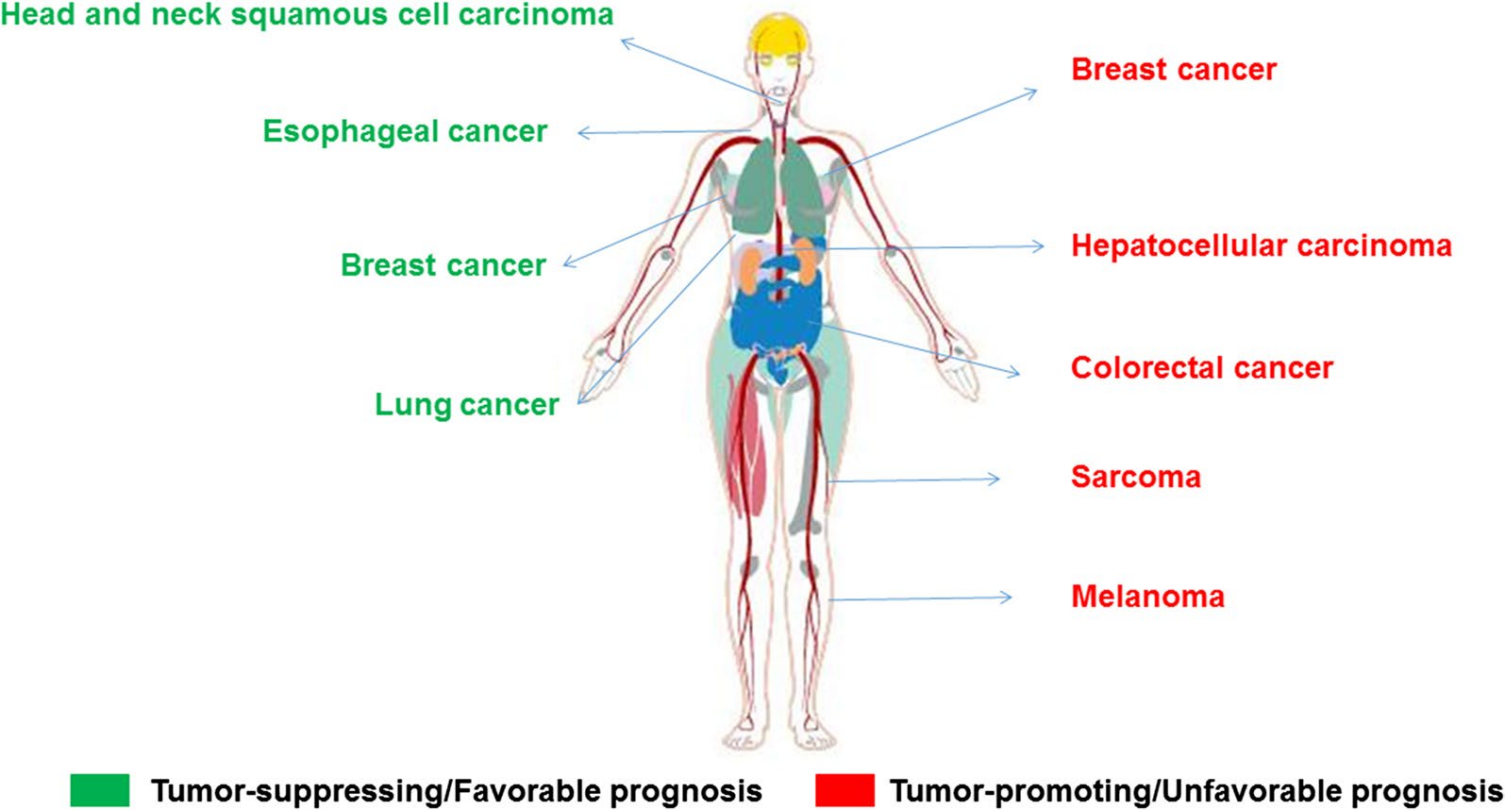
The overview of the roles of USP4 in the cancers mentioned in the present review. USP4 can play a tumor-suppressing role or be favorable prognostic in head and neck squamous cell carcinoma, esophageal cancer, breast cancer and lung cancer and play a tumor-promoting role or be unfavorable prognostic in breast cancer, hepatocellular carcinoma, colorectal cancer, sarcoma and melanoma

The therapeutic and prognostic potential of USP4 in various cancers has been recently revealed in view of numerous regulated pivotal signaling pathways through its deubiquitination capacity. Therefore, aberrant USP4 has been explored in numerous cancers and related to either favorable or unfavorable prognosis of the patients. Survival curve using Kaplan–Meier method based on The Cancer Genome Atlas (TCGA) database (gepia.cancer-pku.cn) in Fig. 2 showed the prognostic potential in several tumor types. The differential expression of USP4 in several tumors’ tissues are significantly related to patients’ overall survival (OS) or disease-free survival (DFS). The overview of the roles of USP4 in cancers mentioned in this review is demonstrated in Table 2 and Fig. 3.

**Table 2:** The substrates and mechanisms of USP4 in cancers mentioned in this review

Authors, year	Type of cancer	In vivo or in vitro study	USP4 expression	Deubiquitinated targets	Effects to the deubiquitinated targets	Regulated tumor biological behaviors	Refs.
Xiao et al., 2012	Lung cancer	In vitro	Down	TRAF2 and TRAF6	Inactivation	Cell migration inhibition	[42]
Mehić et al., 2017	Lung cancer	In vitro	Down	HAS2	Inactivation	–	[88]
Hwang et al., 2016	Lung cancer	In vitro and in vivo	Up	β-catenin	Stabilization	Cell migration and invasion enhancement; Brain metastasis of lung cancer	[90]
Zhang et al., 2012	Breast cancer	In vitro	Up	TβRI	Stabilization	Cell migration enhancement	[27]
Wang et al., 2018	Breast cancer	In vitro and in vivo	Up	HDAC2	Stabilization	Cell proliferation and tumor growth enhancement	[92]
Li et al., 2016	Breast cancer	In vitro and in vivo	Down	PCD4	Stabilization	Cell proliferation and tumor growth inhibition	[94]
Qiu et al., 2018	Liver cancer	In vitro and in vivo	Up	TβRI	Stabilization	Tumor metastasis enhancement	[97]
Li et al., 2018	Liver cancer	In vitro and in vivo	Up	CypA	Stabilization	Tumor growth and metastasis enhancement	[98]
Yun et al., 2015	Colorectal cancer	In vitro	Up	β-catenin	Stabilization	Cell migration and invasion enhancement	[37]
Xing et al., 2015	Colorectal cancer	In vitro and in vivo	Up	PRL-3	Stabilization	Tumor growth and metastasis enhancement	[101]
Hou et al., 2013	Head and neck squamous cell carcinoma	In vitro	Up	RIP1	Inactivation	TNFα-induced cell apoptosis enhancement	[105]

#### Lung cancer

Lung cancer is notorious for its stubbornly high morbidity and mortality which urgently requires effective targeted therapy [85]. USP4 has been reported to play a tumor-suppressing role in lung cancer up to date. Through analyzing TCGA database, USP4 mRNA level was significantly downregulated in lung cancer tissues. And Kaplan–Meier survival analysis confirmed a favorable survival in high USP4 expression group. Besides, USP4 expression was also an independent prognostic factor of OS and recurrence-free survival [86]. The mechanisms of USP4 in lung cancer progression are complicated. Using human lung adenocarcinoma epithelial cell line, A549, Xiao et al. found the tumor-suppressing role of USP4 may result from its negative regulation of NF-κB signaling pathway through targeting K63-linked polyubiquitination of TRAF2 and TRAF6 and inactivate the two proteins, then inhibiting TNFα-induced cancer cell migration [43]. Hyaluronan synthase 2 (HAS2) can promote malignant biological behaviors of cancer through activating various intracellular signaling pathways [87, 88]. Immunohistochemical staining showed a negative correlation between HAS2 and USP4 expression. Mechanically, USP4 mono-deubiquitinates HAS2 which impairs its activity rather than maintaining its stability [89]. When it comes to the reason causing downregulation of USP4 in lung cancer, snail 1 may take the major responsibility and lead to macrophage-dependent inflammation and therapeutic resistance [90]. However, Hwang and his colleague found that upregulated expression of USP4 can facilitate brain metastasis of lung cancer by stabilizing β-catenin expression and knockdown of USP4 showed an increased OS and brain metastasis-free survival which implied an opposite role of USP4 in lung cancer [91].

#### Breast cancer

The role of USP4 in breast cancer progression is complicated and contradictory which depends on different upstream and downstream signaling pathways. In breast cancer cell lines, USP4 was initially found to be phosphorylated by pAKT and exported from nucleus to deubiquitinate and stabilize TβRI and activate downstream TGF-β signaling pathway [28]. Subsequently, Cao et al. further confirmed that USP4 can promote cell migration and invasion through relaxin/TGF-β1/smad2/matrix metalloprotein-9 signaling pathway [92]. The tumor-promoting role of USP4 also relates with HDAC2 activation. USP4 interacted with tricho-rhino-phalangeal syndrome type I to form a complex which led to HDAC2 deubiquitination, then promoted tumor growth both in vitro and in vivo [93]. Higher expression of USP4 is regulated by several molecules in breast cancer. Besides, Geng et al. discovered that a p21-activated kinase 5 (PAK5)/PAK5-aspartyl aminopeptidase (DNPEP)/USP4 axis can be involved in breast cancer progression in which higher level of PAK5 and USP4 expression predicted a worse OS and USP4 can be degradation through hydrolysis pathway regulated by phosphorylated DNPEP [94]. On the contrary, Li et al. found USP4 significantly inhibited breast cancer growth in vivo. Using microarray analysis, programmed cell death 4 (PCD4) signaling pathway was among the most significant upregulation pathways when compared ectopic USP4 expression cell group with control cell group. Further study indicated that USP4 can target and deubiquitinate PCD4 which led to a tumor-suppressing effect [95]. Tumor-suppressing role of USP4 can also be modulated by miRNAs. As aforementioned, miR-148a is not only related to liver fibrosis inhibition, but also to tumor repression in breast cancer by sponging USP4 to hamper its expression [96]. Moreover, miR-553 can also inhibit USP4 expression in breast cancer tissues and the effect can be inversed by ectopic circBMPR2 [97].

#### Liver cancer

TGF-β signaling pathway is involved in the modulation of USP4 to liver cancer progression. USP4 exerted its tumor-promoting role in liver cancer by deubiquitinating and stabilizing TβRI and then activated TβRI/pSmad2 signaling pathway which strengthened cell migration and invasion capacity both in vitro and in vivo [98]. Using quantitative proteomics analysis, cyclophilin A (CypA) was chosen to be another potential target of USP4 in liver cancer which was further confirmed by Co-IP assay. CypA was then demonstrated to mediate malignant biological behaviors, such as cell proliferation and metastasis, caused by USP4 expression [99]. Similar to breast cancer, USP4 can also be negatively regulated by miR-148a which implied the potential therapeutic role of miR-148a in liver cancer [100].

#### Colorectal cancer

As mentioned above, USP4 facilitates colorectal cancer progression by deubiquitinating and stabilizing β-catenin from its degradation which activates its downstream signaling pathways [38]. Subsequently, the same team screened to a highly selective USP4 inhibitor, neural red (NR). NR administration decreased β-catenin stability and hampered tumor growth and metastasis which can be used as an potential anticancer drug in colorectal cancer treatment [101]. USP4 overexpression promoted the level of pAKT in colorectal cancer and this process can be blocked by knockdown of phosphatase of regenerating liver-3 (PRL-3) which can be deubiquitinated and stabilized by USP4 [102]. These studies imply various mechanisms of USP4 in colorectal cancer progression.

#### Other cancers

USP4 knockdown in glioblastoma hindered cell proliferation and induced tumor cell apoptosis through inactivating canonical TGF-β signaling pathway (TβRI/pSmad2) and non-canonical TGF-β signaling pathway (TβRI/phosphorylated extracellular regulated protein kinases1/2). However, the detailed mechanisms remained unknown [103]. USP4 can mediate temozolomide chemoresistance by inhibiting p53 expression. Nevertheless, whether USP4 directly or indirectly regulates p53 expression in glioblastoma still remains unclear [104]. P53 was also shown to mediate USP4 overexpression-inhibited melanoma cell apoptosis without knowing the detailed mechanisms [105]. In head and neck squamous cell carcinoma, USP4 can abrogate receptor-interacting protein 1 (RIP1)-induced NF-κB signaling pathway activation by targeting K63-linked polyubiquitination of RIP1 which promotes TNFα induced cell apoptosis [106]. In esophageal cancer, higher expression of USP4 was observed in tumor tissues than in para-tumor tissues. However, high USP4 expression in tumor tissues was correlated with a favorable OS. And in the small tumor (diameter ≤ 5 cm) and early stage (stages 1 and 2) subgroups, higher USP4 expression was also associated with a better survival [107].

## Conclusion and future perspectives

USP4, as a member of the deubiquitinase family, has extensive substrates to regulate a variety of signaling pathways. USP4 is involved in the regulation of many crucial signaling pathways due to its catalytic activity. Because many pathological and physiological processes are regulated by deubiquitination, USP4 is also involved in these processes and plays complicated regulatory roles, including tumor progression.

The roles of USP4 in tumors are diverse and complex. Due to its diverse downstream substrates and regulatory signaling pathways, USP4 may possess completely opposite effects even in the same tumor. According to previous studies, USP4 mainly plays a tumor-promoting role in liver cancer and colorectal cancer. However, in lung cancer and breast cancer, USP4 possesses completely opposite effects on tumor progression. We can assume that the role of USP4 is tumor-dependent and microenvironment-dependent, and may have different effects in different subtypes of the same tumor. Therefore, the expression level of USP4 in different tumor tissues compared with adjacent para-tumor tissues should be evaluated. The patients whose tumor tissues showed a higher USP4 expression may be more profitable using USP4 specific inhibitors than those have a lower USP4 expression. Given USP4 play a tumor-suppressing role in a few cancers, although USP4 can’t be used as a therapeutic target, exogenous USP4 administration may inhibit the progression of these cancers. Moreover, it can also be used as a favorable prognostic biomarker to distinguish the patients in order to acquire more specific treatment.

In view of the regulatory roles of USP4 in the progression of a variety of tumors, the development of targeted drugs or inhibitors targeting USP4 can be considered as a far-reaching and valuable task. However, although current studies have identified many small molecule inhibitors targeting other members of USP families or nonselective USP4 inhibitors, such as vialinin A and PR619, there have been no studies digging into USP4-specific inhibitors and targeted drugs so far, not to mention clinical trials. The main reason may be that there are many members of the USP family and the domains of these molecules are similar and conserved. It is hard to find a unique domain in USP4 using as a target. Compared to other USPs, the inhibitors developed can target USP4 are fewer. In a previous study of colorectal cancer indicated that NR was found to specifically inhibit tumor growth and metastasis caused by USP4 both in vitro and in vivo, moreover, the effect of NR outshone that of 5-FU. Therefore, in the future, whether NR can also play an excellent anti-cancer effect in other tumors should be further studied. In addition, the focus of future researches should also focus on the discovery of more specific and effective inhibitors that target USP4, and give full play to the therapeutic target functions of USP4.

## Data Availability

Not applicable.

## Abbreviations

ACC: Adrenocortical carcinoma
AKT: Protein kinase B
ARF-BP1: ARF-binding protein 1
BRAC: Breast invasive carcinoma
CARD: Caspase recruitment domain
CDK: Cyclin-dependent kinase
CypA: Cyclophilin A
DNPEP: PAK5-aspartyl aminopeptidase
DUB: Deubiquitylating enzyme
Dvl: Dishevelled
EMT: Epithelial–mesenchymal transition
EV71: Enterovirus 71
Fz: Frizzled
HAS2: Hyaluronan synthase 2
HDAC: Histone deacetylase
HSC: Hepatic stellate cell
ICH: Intracerebral hemorrhage
IFN: Interferon
IL-1R: IL-1 receptor
IRF: Interferon regulatory factor
JNK: c-Jun N-terminal kinase
KICH: Kidney chromophobe
KIRC: Kidney renal clear cell carcinoma
KIRP: Kidney renal papillary cell carcinoma
LEF: Lymphoid enhancer factor
LGG: Brain lower grade glioma
LUAD: Lung adenocarcinoma
NAFLD: Nonalcoholic fatty liver disease
NF-κB: Nuclear factor kappa B
NLK: Nemo-like kinase
NR: Neural red
OS: Overall survival
PAAD: Pancreatic adenocarcinoma
PAK5: p21-activated kinase 5
PCD4: Programmed cell death 4
PRL-3: Phosphatase of regenerating liver-3
PTM: Post-translational modification
RIG-I: Retinoic acid-inducible gene I
RIP1: Receptor-interacting protein 1
RLR: Retinoic acid-inducible gene I-like receptor
RORγt: Retinoic acid-related orphan receptor γt
SART3: Squamous cell carcinoma antigen recognized by T cells 3
TAK1: Transforming growth factor-β-activated kinase 1
TCF: T-cell factor
TCGA: The Cancer Genome Atlas
TGF-β: Tumor growth factor-β
TβRI: TGF-β receptor I
TβRII: TGF-β receptor II
TLR: Toll-like receptor
TNFα: Tumor necrosis factor α
TRAF: TNF receptor associated factor
USP: Ubiquitin-specific protease

## References

[1] Siegel RL, Miller KD, Jemal A (2020). Cancer statistics, 2020. CA Cancer J Clin.

[2] Smith GL, Lopez-Olivo MA, Advani PG, Ning MS, Geng Y, Giordano SH (2019). Financial burdens of cancer treatment: a systematic review of risk factors and outcomes. J Natl Compr Cancer Netw.

[3] May P, Normand C, Morrison RS (2020). Economics of palliative care for cancer: interpreting current evidence, mapping future priorities for research. J Clin Oncol.

[4] Maeda S, Unno M, Yu J (2019). Adjuvant and neoadjuvant therapy for pancreatic cancer. J Pancreatol.

[5] Wu WM, Jin G, Wang CY, Miao Y, Wang HZ, Lou WH, Pancreatic Surgery Study Group of Chinese Society of Surgery of Chinese Medical Association (2019). The current surgical treatment of pancreatic cancer in China: a national wide cross-sectional study. J Pancreatol.

[6] Dezube AR, Jaklitsch MT (2020). New evidence supporting lung cancer screening with low dose CT & surgical implications. Eur J Surg Oncol.

[7] Siegel RL, Miller KD, Jemal A (2020). Cancer statistics, 2019. CA Cancer J Clin.

[8] Liang J, Shi J, Wang N, Zhao H, Sun J (2019). Tuning the protein phosphorylation by receptor type protein tyrosine phosphatase epsilon (PTPRE) in normal and cancer cells. J Cancer.

[9] Balasubramaniyan N, Luo Y, Sun AQ, Suchy FJ (2013). SUMOylation of the farnesoid X receptor (FXR) regulates the expression of FXR target genes. J Biol Chem.

[10] Busold S, Nagy NA, Tas SW, van Ree R, de Jong EC, Geijtenbeek TBH (2020). Various tastes of sugar: the potential of glycosylation in targeting and modulating human immunity via C-type lectin receptors. Front Immunol.

[11] Fu L, Cui CP, Zhang X, Zhang L (2019). The functions and regulation of Smurfs in cancers. Semin Cancer Biol.

[12] Zhu H, Wei T, Cai Y, Jin J (2020). Small molecules targeting the specific domains of histone-mark readers in cancer therapy. Molecules.

[13] Chen B, Sun Y, Niu J, Jarugumilli GK, Wu X (2018). Protein lipidation in cell signaling and diseases: function, regulation, and therapeutic opportunities. Cell Chem Biol.

[14] Brentville VA, Vankemmelbeke M, Metheringham RL, Durrant LG (2020). Post-translational modifications such as citrullination are excellent targets for cancer therapy. Semin Immunol.

[15] Oo HZ, Seiler R, Black PC, Daugaard M (2018). Post-translational modifications in bladder cancer: expanding the tumor target repertoire. Urol Oncol.

[16] Heo KS (2019). Regulation of post-translational modification in breast cancer treatment. BMB Rep.

[17] Li X, Elmira E, Rohondia S, Wang J, Liu J, Dou QP (2018). A patent review of the ubiquitin ligase system: 2015–2018. Expert Opin Ther Pat.

[18] Jang HH (2018). Regulation of protein degradation by proteasomes in cancer. J Cancer Prev.

[19] Veggiani G, Gerpe MCR, Sidhu SS, Zhang W (2019). Emerging drug development technologies targeting ubiquitination for cancer therapeutics. Pharmacol Ther.

[20] Nijman SM, Luna-Vargas MP, Velds A, Brummelkamp TR, Dirac AM, Sixma TK (2005). A genomic and functional inventory of deubiquitinating enzymes. Cell.

[21] Grabbe C, Husnjak K, Dikic I (2011). The spatial and temporal organization of ubiquitin networks. Nat Rev Mol Cell Biol.

[22] Glickman MH, Ciechanover A (2002). The ubiquitin-proteasome proteolytic pathway: destruction for the sake of construction. Physiol Rev.

[23] Young MJ, Hsu KC, Lin TE, Chang WC, Hung JJ (2019). The role of ubiquitin-specific peptidases in cancer progression. J Biomed Sci.

[24] Altun M, Kramer HB, Willems LI, McDermott JL, Leach CA, Goldenberg SJ (2011). Activity-based chemical proteomics accelerates inhibitor development for deubiquitylating enzymes. Chem Biol.

[25] Kapuria V, Peterson LF, Fang D, Bornmann WG, Talpaz M, Donato NJ (2010). Deubiquitinase inhibition by small-molecule WP1130 triggers aggresome formation and tumor cell apoptosis. Cancer Res.

[26] Sacco JJ, Coulson JM, Clague MJ (2010). Emerging roles of deubiquitinases in cancer-associated pathways. IUBMB Life.

[27] Soboleva TA, Jans DA, Johnson-Saliba M, Baker RT (2005). Nuclear-cytoplasmic shuttling of the oncogenic mouse UNP/USP4 deubiquitylating enzyme. J Biol Chem.

[28] Zhang L, Zhou F, Drabsch Y, Gao R, Snaar-Jagalska BE, Mickanin C (2012). USP4 is regulated by AKT phosphorylation and directly deubiquitylates TGF-β type I receptor. Nat Cell Biol.

[29] Deng L, Chen L, Zhao L, Xu Y, Peng X, Wang X (2019). Ubiquitination of Rheb governs growth factor-induced mTORC1 activation. Cell Res.

[30] Das T, Kim EE, Song EJ (2019). Phosphorylation of USP15 and USP4 regulates localization and spliceosomal deubiquitination. J Mol Biol.

[31] Kwon SK, Kim EH, Baek KH (2017). RNPS1 is modulated by ubiquitin-specific protease 4. FEBS Lett.

[32] Uras IZ, List T, Nijman SM (2012). Ubiquitin-specific protease 4 inhibits mono-ubiquitination of the master growth factor signaling kinase PDK1. PLoS ONE.

[33] Zhao B, Velasco K, Sompallae R, Pfirrmann T, Masucci MG, Lindsten K (2012). The ubiquitin specific protease-4 (USP4) interacts with the S9/Rpn6 subunit of the proteasome. Biochem Biophys Res Commun.

[34] Vlasschaert C, Xia X, Gray DA (2016). Selection preserves Ubiquitin Specific Protease 4 alternative exon skipping in therian mammals. Sci Rep.

[35] He TC, Sparks AB, Rago C, Hermeking H, Zawel L, da Costa LT (1998). Identification of c-MYC as a target of the APC pathway. Science.

[36] Tetsu O, McCormick F (1999). Beta-catenin regulates expression of cyclin D1 in colon carcinoma cells. Nature.

[37] Zhao B, Schlesiger C, Masucci MG, Lindsten K (2009). The ubiquitin specific protease 4 (USP4) is a new player in the Wnt signalling pathway. J Cell Mol Med.

[38] Yun SI, Kim HH, Yoon JH, Park WS, Hahn MJ, Kim HC (2015). Ubiquitin specific protease 4 positively regulates the WNT/β-catenin signaling in colorectal cancer. Mol Oncol.

[39] Zhang J, Zhang X, Xie F, Zhang Z, van Dam H, Zhang L (2014). The regulation of TGF-β/SMAD signaling by protein deubiquitination. Protein Cell.

[40] Zhou F, Xie F, Jin K, Zhang Z, Clerici M, Gao R (2017). USP4 inhibits SMAD4 monoubiquitination and promotes activin and BMP signaling. EMBO J.

[41] Fan YH, Yu Y, Mao RF, Tan XJ, Xu GF, Zhang H (2011). USP4 targets TAK1 to downregulate TNFα-induced NF-κB activation. Cell Death Differ.

[42] Liang L, Fan Y, Cheng J, Cheng D, Zhao Y, Cao B (2013). TAK1 ubiquitination regulates doxorubicin-induced NF-κB activation. Cell Signal.

[43] Xiao N, Li H, Luo J, Wang R, Chen H, Chen J (2012). Ubiquitin-specific protease 4 (USP4) targets TRAF2 and TRAF6 for deubiquitination and inhibits TNFα-induced cancer cell migration. Biochem J.

[44] Li Z, Hao Q, Luo J, Xiong J, Zhang S, Wang T (2016). USP4 inhibits p53 and NF-κB through deubiquitinating and stabilizing HDAC2. Oncogene.

[45] Zhou L, Jiang H, Du J, Li L, Li R, Lu J (2018). USP15 inhibits multiple myeloma cell apoptosis through activating a feedback loop with the transcription factor NF-κBp65. Exp Mol Med.

[46] Chen Y, Xin H, Peng H, Shi Q, Li M, Yu J (2020). Hypomethylation-linked activation of PLCE1 impedes autophagy and promotes tumorigenesis through MDM2-mediated ubiquitination and destabilization of p53. Cancer Res.

[47] Song Y, Liu Y, Pan S, Xie S, Wang ZW, Zhu X (2020). Role of the COP1 protein in cancer development and therapy. Semin Cancer Biol.

[48] Fu R, Yang P, Sajid A, Li Z (2019). Avenanthramide A induces cellular senescence via miR-129-3p/Pirh2/p53 signaling pathway to suppress colon cancer growth. J Agric Food Chem.

[49] Chen D, Brooks CL, Gu W (2006). ARF-BP1 as a potential therapeutic target. Br J Cancer.

[50] Zhang X, Berger FG, Yang J, Lu X (2011). USP4 inhibits p53 through deubiquitinating and stabilizing ARF-BP1. EMBO J.

[51] Ellis EL, Mann DA (2012). Clinical evidence for the regression of liver fibrosis. J Hepatol.

[52] Zhu J, Luo Z, Pan Y, Zheng W, Li W, Zhang Z (2019). H19/miR-148a/USP4 axis facilitates liver fibrosis by enhancing TGF-β signaling in both hepatic stellate cells and hepatocytes. J Cell Physiol.

[53] Wu HM, Kim TH, Kim A, Koo JH, Joo MS, Kim SG (2019). Liver X receptor α-induced cannabinoid receptor 2 inhibits ubiquitin-specific peptidase 4 through miR-27b, protecting hepatocytes from TGF-β. Hepatol Commun.

[54] Zhao Y, Gao L, Xu L, Tong R, Lin N, Su Y (2018). Ubiquitin-specific protease 4 is an endogenous negative regulator of metabolic dysfunctions in nonalcoholic fatty liver disease. Hepatology.

[55] Zhou J, Qiu T, Wang T, Chen Z, Ma X, Zhang L (2019). USP4 deficiency exacerbates hepatic ischaemia/reperfusion injury via TAK1 signalling. Clin Sci.

[56] Akira S, Takeda K (2004). Toll-like receptor signalling. Nat Rev Immunol.

[57] Zhou F, Zhang X, van Dam H, Ten Dijke P, Huang H, Zhang L (2012). Ubiquitin-specific protease 4 mitigates Toll-like/interleukin-1 receptor signaling and regulates innate immune activation. J Biol Chem.

[58] Yang J, Xu P, Han L, Guo Z, Wang X, Chen Z (2015). Cutting edge: ubiquitin-specific protease 4 promotes Th17 cell function under inflammation by deubiquitinating and stabilizing RORγt. J Immunol.

[59] Lee W, Kim HS, Baek SY, Lee GR (2016). Transcription factor IRF8 controls Th1-like regulatory T-cell function. Cell Mol Immunol.

[60] Vignali DA, Collison LW, Workman CJ (2008). How regulatory T cells work. Nat Rev Immunol.

[61] Lin R, Nie J, Ren J, Liang R, Li D, Wang P (2017). USP4 interacts and positively regulates IRF8 function via K48-linked deubiquitination in regulatory T cells. FEBS Lett.

[62] Guo Z, Xu P, Ge S, Zhang C, Zheng X, Xu J (2017). Ubiquitin specific peptidase 4 stabilizes interferon regulatory factor protein and promotes its function to facilitate interleukin-4 expression in T helper type 2 cells. Int J Mol Med.

[63] Liu C, Liu C, Liu H, Gong L, Tao T, Shen Y (2017). Increased expression of ubiquitin-specific protease 4 participates in neuronal apoptosis after intracerebral hemorrhage in adult rats. Cell Mol Neurobiol.

[64] Jiang X, Yu M, Ou Y, Cao Y, Yao Y, Cai P (2017). Downregulation of USP4 promotes activation of microglia and subsequent neuronal inflammation in rat spinal cord after injury. Neurochem Res.

[65] Zhang L, Zhao X, Zhang M, Zhao W, Gao C (2014). Ubiquitin-specific protease 2b negatively regulates IFN-β production and antiviral activity by targeting TANK-binding kinase 1. J Immunol.

[66] Pauli EK, Chan YK, Davis ME, Gableske S, Wang MK, Feister KF (2014). The ubiquitin-specific protease USP15 promotes RIG-I-mediated antiviral signaling by deubiquitylating TRIM25. Sci Signal.

[67] Loo YM, Gale M (2011). Immune signaling by RIG-I-like receptors. Immunity.

[68] Oshiumi H, Matsumoto M, Hatakeyama S, Seya T (2009). Riplet/RNF135, a RING finger protein, ubiquitinates RIG-I to promote interferon-beta induction during the early phase of viral infection. J Biol Chem.

[69] Cui J, Song Y, Li Y, Zhu Q, Tan P, Qin Y (2014). USP3 inhibits type I interferon signaling by dubiquitinating RIG-I-like receptors. Cell Res.

[70] Chen R, Zhang L, Zhong B, Tan B, Liu Y, Shu HB (2010). The ubiquitin-specific protease 17 is involved in virus-triggered type I IFN signaling. Cell Res.

[71] Wang L, Zhao W, Zhang M, Wang P, Zhao K, Zhao X (2013). USP4 positively regulates RIG-I-mediated antiviral response through deubiquitination and stabilization of RIG-I. J Virol.

[72] Xu C, Peng Y, Zhang Q, Xu XP, Kong XM, Shi WF (2018). USP4 positively regulates RLR-induced NF-κB activation by targeting TRAF6 for K48-linked deubiquitination and inhibits enterovirus 71 replication. Sci Rep.

[73] Liu H, Zhang H, Wang X, Tian Q, Hu Z, Peng C (2015). The deubiquitylating enzyme USP4 cooperates with CtIP in DNA double-strand break end resection. Cell Rep.

[74] Wijnhoven P, Konietzny R, Blackford AN, Travers J, Kessler BM, Nishi R (2015). USP4 auto-deubiquitylation promotes homologous recombination. Mol Cell.

[75] Gong Y, Slee RB, Fukai N, Rawadi G, Roman-Roman S, Reginato AM (2001). LDL receptor-related protein 5 (LRP5) affects bone accrual and eye development. Cell.

[76] Boyden LM, Mao J, Belsky J, Mitzner L, Farhi A, Mitnick MA (2002). High bone density due to a mutation in LDL-receptor-related protein 5. N Engl J Med.

[77] Liu B, Wu S, Han L, Zhang C (2015). β-catenin signaling induces the osteoblastogenic differentiation of human pre-osteoblastic and bone marrow stromal cells mainly through the upregulation of osterix expression. Int J Mol Med.

[78] Gaur T, Lengner CJ, Hovhannisyan H, Bhat RA, Bodine PV, Komm BS (2005). Canonical WNT signaling promotes osteogenesis by directly stimulating Runx2 gene expression. J Biol Chem.

[79] Glass DA, Bialek P, Ahn JD, Starbuck M, Patel MS, Clevers H (2005). Canonical Wnt signaling in differentiated osteoblasts controls osteoclast differentiation. Dev Cell.

[80] Kramer I, Halleux C, Keller H, Pegurri M, Gooi JH, Weber PB (2010). Osteocyte Wnt/beta-catenin signaling is required for normal bone homeostasis. Mol Cell Biol.

[81] Zhou F, Li F, Fang P, Dai T, Yang B, van Dam H (2016). Ubiquitin-specific protease 4 antagonizes osteoblast differentiation through dishevelled. J Bone Miner Res.

[82] Rudnicki MA, Jaenisch R (1995). The MyoD family of transcription factors and skeletal myogenesis. BioEssays.

[83] Yun SI, Kim KK (2017). Ubiquitin-specific protease 4 (USP4) suppresses myoblast differentiation by down regulating MyoD activity in a catalytic-independent manner. Cell Signal.

[84] He B, Zhao YC, Gao LC, Ying XY, Xu LW, Su YY (2016). Ubiquitin-specific protease 4 is an endogenous negative regulator of pathological cardiac hypertrophy. Hypertension.

[85] Okamoto I (2017). Combination therapy with molecularly targeted agents in lung cancer. Ann Oncol.

[86] Zhong M, Jiang Q, Jin R (2018). USP4 expression independently predicts favorable survival in lung adenocarcinoma. IUBMB Life.

[87] Okuda H, Kobayashi A, Xia B, Watabe M, Pai SK, Hirota S (2012). Hyaluronan synthase HAS2 promotes tumor progression in bone by stimulating the interaction of breast cancer stem-like cells with macrophages and stromal cells. Cancer Res.

[88] Bernert B, Porsch H, Heldin P (2011). Hyaluronan synthase 2 (HAS2) promotes breast cancer cell invasion by suppression of tissue metalloproteinase inhibitor 1 (TIMP-1). J Biol Chem.

[89] Mehić M, de Sa VK, Hebestreit S, Heldin CH, Heldin P (2017). The deubiquitinating enzymes USP4 and USP17 target hyaluronan synthase 2 and differentially affect its function. Oncogenesis.

[90] Lai CY, Yeh DW, Lu CH, Liu YL, Chuang YC, Ruan JW (2020). Epigenetic silencing of ubiquitin specific protease 4 by snail1 contributes to macrophage-dependent inflammation and therapeutic resistance in lung cancer. Cancers.

[91] Hwang SJ, Lee HW, Kim HR, Lee H, Shin CH, Yun SI (2016). Ubiquitin-specific protease 4 controls metastatic potential through β-catenin stabilization in brain metastatic lung adenocarcinoma. Sci Rep.

[92] Cao WH, Liu XP, Meng SL, Gao YW, Wang Y, Ma ZL (2016). USP4 promotes invasion of breast cancer cells via Relaxin/TGF-β1/Smad2/MMP-9 signal. Eur Rev Med Pharmacol Sci.

[93] Wang Y, Zhang J, Wu L, Liu W, Wei G, Gong X (2018). Tricho-rhino-phalangeal syndrome 1 protein functions as a scaffold required for ubiquitin-specific protease 4-directed histone deacetylase 2 de-ubiquitination and tumor growth. Breast Cancer Res.

[94] Geng N, Li Y, Zhang W, Wang F, Wang X, Jin Z (2020). A PAK5-DNPEP-USP4 axis dictates breast cancer growth and metastasis. Int J Cancer.

[95] Li Y, Jiang D, Zhang Q, Liu X, Cai Z (2016). Ubiquitin-specific protease 4 inhibits breast cancer cell growth through the upregulation of PDCD4. Int J Mol Med.

[96] Zhang L, Xing M, Wang X, Cao W, Wang H (2017). MiR-148a suppresses invasion and induces apoptosis of breast cancer cells by regulating USP4 and BIM expression. Int J Clin Exp Pathol.

[97] Liang Y, Song X, Li Y, Ma T, Su P, Guo R (2019). Targeting the circBMPR2/miR-553/USP4 axis as a potent therapeutic approach for breast cancer. Mol Ther Nucleic Acids.

[98] Qiu C, Liu Y, Mei Y, Zou M, Zhao Z, Ye M (2018). Ubiquitin-specific protease 4 promotes metastasis of hepatocellular carcinoma by increasing TGF-β signaling-induced epithelial–mesenchymal transition. Aging.

[99] Li T, Yan B, Ma Y, Weng J, Yang S, Zhao N (2018). Ubiquitin-specific protease 4 promotes hepatocellular carcinoma progression via cyclophilin A stabilization and deubiquitination. Cell Death Dis.

[100] Heo MJ, Kim YM, Koo JH, Yang YM, An J, Lee SK (2014). microRNA-148a dysregulation discriminates poor prognosis of hepatocellular carcinoma in association with USP4 overexpression. Oncotarget.

[101] Nguyen HH, Kim T, Nguyen T, Hahn MJ, Yun SI, Kim KK (2019). A selective inhibitor of ubiquitin-specific protease 4 suppresses colorectal cancer progression by regulating β-catenin signaling. Cell Physiol Biochem.

[102] Xing C, Lu XX, Guo PD, Shen T, Zhang S, He XS (2016). Ubiquitin-specific protease 4-mediated deubiquitination and stabilization of PRL-3 is required for potentiating colorectal oncogenesis. Cancer Res.

[103] Zhou Y, Liang P, Ji W, Yu Z, Chen H, Jiang L (2019). Ubiquitin-specific protease 4 promotes glioblastoma multiforme via activating ERK pathway. Onco Targets Ther.

[104] Qin N, Han F, Li L, Ge Y, Lin W, Wang J (2019). Deubiquitinating enzyme 4 facilitates chemoresistance in glioblastoma by inhibiting P53 activity. Oncol Lett.

[105] Guo W, Ma J, Pei T, Zhao T, Guo S, Yi X (2018). Up-regulated deubiquitinase USP4 plays an oncogenic role in melanoma. J Cell Mol Med.

[106] Hou X, Wang L, Zhang L, Pan X, Zhao W (2013). Ubiquitin-specific protease 4 promotes TNF-α-induced apoptosis by deubiquitination of RIP1 in head and neck squamous cell carcinoma. FEBS Lett.

[107] Yao R, Pu J, Fan R, Zhu W, Ding X, Shen X (2017). Ubiquitin-specific protease 4 improves the prognosis of the patients in esophageal cancer. Cancer Biomark.

